# Some Benchmark Searches for Testing Search Capabilities and Medical Coverage of Internet Discovery Tools

**DOI:** 10.2196/jmir.2.3.e19

**Published:** 2000-09-27

**Authors:** E Diane Johnson

**Keywords:** Internet, Information Storage and Retrieval, Search Engines

## Abstract

The past few years have seen a proliferation of search engines for the World Wide Web (WWW), as well as a growing number of specialized subject directories geared to the needs of health care professionals. Yet documentation on scope, coverage, and search features is often uneven at best; and even documented search features may not perform as advertised. This paper will present a group of sample searches to assist users in gauging database size, determining default search operators, and testing for the presence of advanced search features such as case sensitivity, stemming, and concept mapping for medical topics on English-language web sites.

## Introduction

The software used by World Wide Web (WWW) search engines continues to evolve so rapidly that keeping abreast of search features is a never-ending task. Search engines, such as Northern Light, HotBot, and AltaVista, undergo constant overhauls. Search engine software changes so frequently that help screens, if they exist at all, are often inadequate. At worst, the help screens may even refer to a previous version of the software. Often, even a system with accurate and up-to-date documentation will present it in a Frequently Asked Question and answer format (FAQ), making it difficult to locate specific facts and features of a search engine.

To further add to the confusion, sometimes search engines do not perform as advertised. In other cases, search engines seem to be deliberately vague about the inner workings of their searching software or ranking algorithms because they consider that information to be proprietary.

## Benchmarking Tests for Search Engine Features

### Default Operator

Understanding how an engine combines search terms is essential to effective searching. Many of the search engines, especially in basic or novice mode, employ fuzzy logic, where all search terms are linked with a Boolean OR. This is sometimes referred to as "Match any of the terms." This accounts for the large number of results returned from some search engines.

It is possible to determine the default operator by performing a few simple searches. First, enter a single search term and record the retrieval: a recent search for the word "ear" on Excite retrieved 129,711 pages. Then add a second word to the search: searching "ear infections" on Excite yielded 183,650 pages. Since the second search retrieved more than the first search, the default operator on Excite is OR.

If the second search retrieves a smaller number of results than the first one, the default operator may be AND. Searching "ear" on Northern Light retrieved 959,152 pages, while "ear infections" retrieved 55,560. The smaller results indicates that the default operator may be AND; however, it could also be doing an even narrower search, retrieving only pages with the exact phrase "ear infections." To determine whether the default operator is AND or ADJacency, do a third search with the two terms reversed: "infections ear." If the retrieval is the same as in the second search, as it is for Northern Light, the default operator is almost sure to be AND. If the search result is different, the default is probably an adjacency operator, or an exact phrase search.

### Stopwords

Stopwords, or noise words, can also be problematic in searching. Some search engines index even the smallest words, including "a" and "the." Others have a list of stopwords that are not indexed; these lists are often unpublished. One way to test for the presence of stopwords is to do searches for "vitamin a deficiency" and ""vitamin k deficiency" and compare retrieval. In Excite, both of these searches retrieve 96,794 items, indicating that single letters are indeed stopwords. Another test for stopwords is to enter the search term alone; and indeed a search for "a" in Excite returns no results.

### Database Size

One area where the search engines seem especially prone to hyperbole is in their claims to database size. Here are some claims that have appeared on search engine web pages, either now or in the past:

"Excite Search, the Internet's most comprehensive search tool..."

"AltaVista gives you access to the largest Web index..."

From Hotbot: "...the largest and most complete index of Internet documents in the world."

More than one search engine boasts that its database is the largest and most complete on the WWW. Even when actual numbers on database size are provided, they can be misleading and difficult to compare. One search engine may claim that its database has the most URLs; but this number may be artificially inflated if the database contains many duplicates. And how many of these URLs represent pages which no longer exist? Another search engine may base its claim to be the largest on the size of its database in terabytes [1]. But this may reflect an inefficient file structure more than anything else. How, then, can one accurately gauge the size of a search engine database? Perhaps it is best to focus on estimating relative size based upon retrieval when compared to other search engines. This is easily done by performing benchmark searches for the same word on several different search engines, then comparing the results. A single, unambiguous word works best, one which adequately represents a single concept without a lot of synonyms or variant endings, such as "arthritis." Avoid words which are not specific to the medical domain, which convey a different meaning in a non-medical context; such as "labor," which is used to refer both to childbirth and work (in addition to having an alternative British spelling). As a first step toward gauging the amount of content geared toward health professionals as opposed to patients and health consumers, select a word which is more likely to be used by health practitioners, such as "splenomegaly," "diaphoresis," "dyspnea," "osteoarthritis," or "lymphadenopathy" [see Table 1].

**Table 1:** Results of Benchmark Searches for Medical Terms, Spring 1999

	**Excite**	**HotBot**	**AltaVista**	**MedHunt**	**Medical World Search**
Arthritis	40,191	138,080	311,810	1,992	3,261
Splenomegaly	751	185	194	123	471

One word of warning: determining the number of hits retrieved on a web search is not always easily done. In Excite, one must scroll down the page to reveal the number of hits. When searching a highly posted term in HotBot, the number of hits doesn't appear on the first page of results, only on subsequent pages. Sometime in 1998, Lycos removed the number of hits retrieved entirely from their screens, leaving no way to assess relative size using benchmark searches like these. It is also revealing to compare retrieval for medical terms in some of the larger medical directories with search engine results. Medical World Search, with its database of "nearly 100,000" pages [2], is only about 1/10 of a percent (.1%) the size of the largest search engines like HotBot and AltaVista, which are estimated to index between 100,000,000 - 150,000,000 pages. Yet, even though it is 1,000 times larger overall, AltaVista retrieves only four times as many pages containing "splenomegaly;" HotBot actually retrieves fewer pages than Medical World Search on this term.

### Case Sensitivity

Often the presence of capital letters, or a combination of upper and lower case letters, conveys a specific meaning for a health sciences term. When searching for information on "AIDS," as in Acquired Immunodeficiency Syndrome, a searcher does not want to also retrieve information on hearing "aids." Typically if a search engine recognizes case, it will retrieve both upper and lower case in response to a lower case query (e.g. aids or AIDS), but only upper case if the query is entered that way (e.g. only AIDS). To test for case sensitivity, search for the same word twice: once in upper and once in lower case, and compare the results. If the same number of items is retrieved on both searches, the search engine is not case sensitive.

Performing one additional search will test for the ability to search for terms which contain only a special combination of capital and small letters; this is sometimes referred to as "interesting case." An example of interesting case from the medical domain would be MeSH, referring to the Medical Subject Headings published by the National Library of Medicine. In HotBot, a search for "mesh" retrieves 175,950 items; "MESH" retrieves 7180; but "MeSH" retrieves 5480.

### Stemming

With most search engines, what you type is what you get; nothing more, and nothing less. The engine does a literal search for exactly what is entered. There are two possible exceptions to this: stemming and concept searching.

A search engine which uses stemming will automatically retrieve some words with variant endings. In its simplest form, this operates as automatic right truncation, where a search for "germ" also retrieves "germs," "germinate," and even "Germany." Yahoo uses this type of stemming. Other search engines stem more selectively, perhaps where searching a singular word also retrieves the plural form; e.g. searching "child" retrieves "children," but not "childhood." To test for the first type of stemming via automatic right truncation, search on a word stem such as "occlu" to see if "occlusion," "occluded," etc. are retrieved. The second type of stemming is more difficult to evaluate. Search for a simple plural with and without the "s," then perform the search using both terms linked with OR: first search "kidney," second "kidneys," then "kidney OR kidneys." If all three searches return the same number of hits, simple stemming of singular and plural word forms is in operation. To test for more sophisticated stemming, try an irregular plural: woman vs. women, child vs. children, person vs. people. If results are the same, the stemming is more sophisticated.

### Concept Searching

Some of the search engines, notably Excite and Magellan, claim to be able to conduct concept searches. The user types in a single word, and the search engine purports to search not only that specific word, but also to automatically include synonyms in the search. Unfortunately, this feature is not always optimized for medical terms. One way to tell is by searching on a word such as "kidney," recording the result, and then searching a medical synonym such as "renal," recording that result, and then pooling the two by searching "kidney OR renal." If the last search retrieves many more items than either the first or the second search, one can surmise that concept mapping is weak or perhaps nonexistent in the area of medical vocabulary. Table 2 shows the results of this test in Magellan and Excite, both of which purport to use mapping or ICE (Intelligent Concept Extraction). From these results, ICE apparently is not automatic for terms in the medical domain. However, along with the results, Excite returns a suggestion to add the following words to the search: kidneys, dialysis, nephrology, glomerular, polycystic, ureter, transplant, creatinine, tubule, and nephropathy. But it does not perform automatically as advertised in the help screens, which state:

Excite searches for documents containing the exact words that you enter into the Search box. But that's not all. Excite takes search technology one step further: Not just words, Excite also searches for ideas closely related to the words in your query.

For example, suppose you search on the terms "elderly people financial concerns." In addition to finding sites containing those exact words, the search engine will find sites mentioning the economic status of retired people and the financial concerns of senior citizens [3].

**Table 2:** Results of Tests for Concept Mapping in Magellan and Excite, Spring 1999

	**Magellan**	**Excite**
Renal	1,354	28,494
Kidney	2,349	49,424
Renal OR Kidney	2,509	67,223

One interesting footnote: Excite and Magellan use almost exactly the same wording and examples when explaining their concept search feature, although the results of these sample search illustrate that the two engines perform quite differently. The only way to account for this, although it doesn't really explain it, is that Excite now owns Magellan, even though the latter is still run as a separate search service with its own look, feel, and capabilities.

There is one specialized search engine targeted to a medical audience with relatively sophisticated concept mapping capabilities: Medical World Search (http://www.mwsearch.com). A search of its 100,000 item database of major medical sites retrieves 762 items regardless of whether "acetaminophen" or "tylenol" is searched, since queries are enhanced with terms from the National Library of Medicine's Unified Medical Language System Metathesaurus [4]. Indeed, the search also incorporates "Acetamidophenol," "Acetominophen," "Anacin-3," "Datril," "Hydroxyacetanilide," "N-Acetyl-p-aminophenol," "p-Acetamidophenol," "p-Hydroxyacetanilide," "Panadol," "Paracetamol," "Acamol," "Acetamide, N-(4-hydroxyphenyl)-," and "N-(4-Hydroxyphenyl)acetanilide."

Two words of caution apply when applying these benchmark searches. First, they are simply heuristics for determining search engine behavior, and will not provide definitive evidence of the presence or absence of search features in all situations. Second, if these benchmark searches are run during a database update, results may differ by only one or two hits. For example, one evening, when testing AltaVista for case sensitivity, "aids or AIDS" retrieved only two more hits than a search for "aids" alone had only 5 minutes before. It turned out that these represented two new URLs just added to the database. This was confirmed by re-executing the original search for "aids" alone, which then retrieved two more items than it had just minutes before.

These same techniques can be used to evaluate the search capabilities of the free MEDLINE sites on the Web [5]. For example, while the HealthGate help screens clearly state that drug trade and generic names are mapped to one another [6], a search for the trade name "valium" retrieved 606 items, while a search for "diazepam," the generic name of the same drug, retrieved 6% more: 954 items.

These benchmark searches evolved partly as an byproduct of the Nothing But 'Net website [7], an internet search assistant developed at the J. Otto Lottes Health Sciences Library, University of Missouri - Columbia with the assistance of a grant from the National Network of Libraries of Medicine/Midcontinental Region [8]. The user completes a form selecting the capabilities needed for a given search, e.g. case sensitivity, proximity search, nesting, etc. The request is translated into a query, which is sent to a SQL database that contains information on the features of 15 search engines, including HotBot, AltaVista, Yahoo, MedHunt, and Medical Matrix. The result is a listing of up to three search engines best suited to those types of queries, along with syntax examples. Both full and partial matches are included, with the best match(es) appearing first. For example if the user requested the features "nesting," "within # of words," and "date searching," AltaVista, which supports all 3 search features, is listed first. HotBot and LycosPro are listed next, since they contain 2 out of the 3 search features requested. Help screens are available for each search feature, along with medically related search examples. Nothing But Net, which is updated semi-annually, can be found at: http://hansel.mig.missouri.edu/engines.

## Appendix 1

### Default Operator:

a=ear

b=ear infections

c=infections ear

If b>a, default operator is OR.

If b<a and b=c, default operator is AND.

If b<a and b≠c, default operator is ADJ.

### Database Size:

Benchmark searches:

Arthritis, splenomegaly

### Stopwords

a=vitamin a deficiency

b=vitamin k deficiency

If a=b, "a" is probably a stopword; if a search for "a" or "k" alone yields 0, they are stopwords.

### Case Sensitivity:

a=AIDS

b=aids

If a=b, search engine is not case sensitive.

### Interesting Case:

a=MeSH

b=mesh

c=MESH

If a≠b≠c, searches for interesting case are supported.

### Stemming:

If a search for occlu retrieves occlusion, occlusive, occluded, etc, automatic right truncation is in use.

a=kidney

b=kidneys

If a=b=(a OR b), stemming is enabled for simple plurals.

a=women

b=woman

If a=b, more sophisticated plural stemming is in use.

### Concept Searching:

a=kidney

b=renal

If a >= (a OR b), some concept mapping is taking place.

a=tylenol

b=acetaminophen

If a=b, some concept mapping is in place.
